# Of volatiles and peptides: in search for MHC-dependent olfactory signals in social communication

**DOI:** 10.1007/s00018-014-1559-6

**Published:** 2014-02-05

**Authors:** Peter Overath, Theo Sturm, Hans-Georg Rammensee

**Affiliations:** 1 Interfakultäres Institut für Zellbiologie, Abteilung Immunologie, Universität Tübingen, Auf der Morgenstelle 15, 72076 Tübingen, Germany; 2 Present Address: Eidgenössische Technische Hochschule Zürich (ETH), Institut für Molekulare Systembiologie, Auguste-Piccard-Hof 1, 8093 Zürich, Switzerland

**Keywords:** Single amino acid variation, Polymorphism, Individual recognition, Mouse, Urine, Vomeronasal organ, Sensory neuron

## Abstract

Genes of the major histocompatibility complex (MHC), which play a critical role in immune recognition, are considered to influence social behaviors in mice, fish, humans, and other vertebrates via olfactory cues. As studied most extensively in mice, the polymorphism of MHC class I genes is considered to bring about a specific scent signature, which is decoded by the olfactory system resulting in an individual-specific reaction such as mating. On the assumption that this signature resides in volatiles, extensive attempts to identify these MHC-specific components in urine failed. Alternatively, it has been suggested that peptide ligands of MHC class I molecules are released into urine and can elicit an MHC-haplotype-specific behavioral response after uptake into the nose by sniffing. Analysis of the urinary peptide composition of mice shows that MHC-derived peptides are present, albeit in extremely low concentrations. In contrast, urine contains abundant peptides which differ between mouse strains due to genomic variations such as single-nucleotide variations or complex polymorphisms in multigene families as well as in their concentration. Thus, urinary peptides represent a real-time sampling of the expressed genome available for sensory evaluation. It is suggested that peptide variation caused by genomic differences contains sufficient information for individual recognition beyond or instead of an influence of the MHC in mice and other vertebrates.

## Introduction

The hypothesis that there is a connection between the immune system and social behaviors such as mate choice mediated via olfactory cues is now nearly four decades old [1]. This idea has not only fascinated numerous scientists stimulating an extensive number of experimental and theoretical studies but at the same time found entry as an established fact into public knowledge. Specifically, it was proposed that the major histocompatibility complex (MHC) involved in the adaptive immune response is in addition responsible for the production of characteristic chemical signals in bodily secretions that enable individual recognition in social contexts such as mate or kin recognition by olfaction [2–5]. The MHC, designated H2 in mice and HLA in humans, is a genomic region on chromosomes 17 and 6, respectively, which harbors the highly polymorphic MHC class I and class II genes as well as many non-polymorphic genes [6]. The specificity of the olfactory signals is considered to reflect the variable structure of the plasma membrane-associated proteins encoded by the MHC class I and II genes. Although MHC social signaling is thought to occur in over 20 species of vertebrates [5], studies into its molecular mechanism have been predominantly performed with mice and more recently in fish and man [7, 8]. This review will mainly concentrate on studies in mice, but will also summarize studies in fish and man. We will briefly discuss the experiments that have suggested the involvement of the MHC in social communication and then describe the quest for the identification of MHC-dependent chemical signals with social meaning. We will conclude that there is no convincing evidence that the MHC influences the bouquet of volatiles from mouse urine. Furthermore, the urinary peptide composition is only marginally modulated by the MHC. Instead, we propose that genomic differences and post-genomic effects on the peptidome provide ample variability that can be used as information for the sensory evaluation of individuality.

## Relating MHC and social interaction in mice

In now-classical mating experiments, a male inbred mouse of one MHC type (e.g., B6-H2^b^) was given the choice between two congenic females, one with the same MHC (B6-H2^b^) and the other with a different MHC (B6-H2^k^) on the same B6 genetic background. The males showed a statistically significant preference (about 70 % compared to a random 50 %) to mate with the female carrying the alternative MHC (H-2^k^, disassortative mating). This strain preference was confirmed in matings involving homozygous H2^b^ and H2^k^ F_2_ segregants of the two congenic strains. In the reverse situation, where a male mouse of B6-H2^k^ genotype had to choose between the two congenic females, there was a similar bias for the female with the other MHC (H2^b^), however this preference disappeared in matings of F_2_ segregants [2, 9]. Disassortative mating preference has been observed in other congenic strain combinations but there are also examples with no strain preference or an assortative bias, i.e., the male favored the female with its own rather than the different MHC [9, 10]. If newborn B6-H2^b^ males were fostered on B6-H2^k^ parents, grown-up males now preferred to mate with B6-H2^b^ females. This indicates that differential mating is not an innate but rather a learned response, which depends on the parental environment in early life [11, 12]. MHC-associated non-random matings have also been demonstrated in seminatural populations of mice in that 27 % fewer MHC-homozygous offspring were observed than expected from random mating [13].

Assuming that disassortative mating is based on differences in volatile olfactory cues, an extensive series of studies using a Y-maze was initiated. In such a device, differentially scented currents of air were drawn from odor chambers and conducted through the two arms of the Y. Although sensor mice showed no spontaneous MHC preference when smelling the odor of MHC-congenic pairs of males, they could be trained to distinguish the scent of these strains or of MHC-congenic F_2_-segregants derived thereof (performance score of about 80 % against a 50 % score by chance, cf. reference [14]). Importantly, the body odor could be replaced by volatiles released from corresponding urine samples, suggesting that urine contains the critical MHC-dependent components [15, 16]. Furthermore, studies on mutants with changes in the peptide-binding region of MHC class I products or the use of β_2_m knockout mice, which lack the expression of functional MHC class I molecules, indicated that the profile of urine-derived volatiles might be directly or indirectly influenced by this group of highly polymorphic proteins [17, 18]. Training in the Y maze involved solely recognition by the main olfactory epithelium (MOE) because the rate of learning to differentiate the urinary volatiles of B6-H2^b^ and B6-H2^k^ strains was not influenced by the ablation of the vomeronasal organ (VNO, reference [19]). Behavioral tests not involving training but differential sniffing at urine samples supported the notion that classical MHC genes influence the individual scent. In this assay both, the MOE and the VNO were activated and the relevant components could be volatile or non-volatile [20, 21].

While the training studies supported the idea that the MHC influences the composition of urinary scent, other investigators [22] argued that establishing the difference between two scents is not sufficient evidence for demonstrating individual recognition. Rather, this requires a specific behavioral response such as competitive scent marking, which involves activation of the VNO. In this assay, a mark of a urine mixture from males of one mouse strain was deposited on adsorbent paper, which was then introduced into the territory of a resident mouse. The number of urinary marks of the resident mouse deposited on the paper was taken as a measure of the novelty of the donor’s urine scent. Differences in H2 of congenic strains could be discriminated as evidenced by close contact investigation of urine marks but failed to stimulate a countermarking response. Thus, neither volatile nor non-volatile odors influenced by the MHC were required or sufficient to elicit competitive scent marking, which, however, was clearly observed between unrelated inbred strains. Additional studies showed that wild-derived female mice did not use differences in MHC type of competitor males for individual recognition [23].

Another functional test for individual recognition is the selective pregnancy block (Bruce effect, cf. reference [24]). This assay has been used to test the effect of synthetic peptides known to be presented by MHC class I molecules [25]. If a female mouse, e.g., of inbred strain BALB/c, is mated with a B6 male, subsequent exposure to a male of a different strain, for example a BALB/c mouse, or its urine led to implantation failure and abortion of her mate’s offspring. During mating, the female forms an enduring memory of the individual identity of the mating male’s chemosignals via the vomeronasal system [26]. For BALB/c females mated to B6 males, it was shown that peptide ligands with anchor residues typical for BALB/c MHC class I molecules could elicit the pregnancy block when added to B6 urine while addition of ligands typical for the B6 MHC class I molecules to B6 urine was ineffective. Thus, MHC peptide ligands of the non-mating male but not of the mating male MHC type could convert familiar to strange urine. These experiments implied that mouse urine contains MHC-derived peptides, which are important for the formation of an olfactory recognition memory in the context of the pregnancy block [25].

In summary, several but not all behavioral studies suggest that the MHC or, more specifically, MHC class I genes directly or indirectly influence the composition of urinary volatiles or peptides or both. Correlations between differences in urine composition and variation in H2 are a first step in unraveling the molecular mechanism of MHC in social communication beyond its function in the immune system.

## The search for MHC-dependent volatiles in mouse urine

Urinary scent of male mice contains hundreds of volatile components: aliphatic acids, ketones, aldehydes, alcohols, amines, and thiols, which vary widely in concentration. Important constituents are putative signaling molecules such as 6-hydroxy-6-methyl-3-heptanone, 2-heptanone, 2, 3-dehydro-exobrevicomin, 2-sec-butyl-4,5-dihydrothiazole, *E*-β-farnesene, (*E,E*)-α-farnesene, trimethylamine and (methylthio)methanethiol (cf. reviews references [27, 28]). Male urine contains high concentrations of major urinary proteins (MUPs), which bind components such as 2,3-dehydro-exobrevicomin and 2-sec-butyl-4,5-dihydrothiazole, and thereby extend the duration of ligand transfer to the gas phase. As shown for male mice of the C57BL/6J strain, the composition of urinary scent and body scent differs significantly [29]. From a total of 67 components, nine were observed only in the volatiles from the body of mice. On the other hand, 20 substances including 2, 3-dehydro-exo-brevicomin were only found in urinary volatiles. All other substances were present in the volatiles of both mice and their urine. Aliphatic aldehydes from pentanal to decanal were prominent mouse body odor components. Because receptors for these aldehydes have been extensively characterized in the main olfactory organ [30, 31], these components may be important for mice in recognizing their conspecifics from a distance.

A recent review [32] lists a dozen studies performed over three decades aimed at identifying urinary volatiles that can be correlated to the MHC in inbred, congenic, and mutant mice. Considerable differences regarding the components detected in various laboratories were reported. These differences largely reflect the distinct methodologies used in volatile collection ranging from older purge and trap procedures to more recently developed techniques of sorptive extraction to hydrophobic polymers in the gas or liquid phase. Most importantly, no volatiles have been identified that differ consistently between MHC congenic strains. In contrast, all studies reported quantitative differences in component abundance, which appear to be regulated by MHC variation. However, no consensus was reached concerning the type, number, and amount of components that differ quantitatively.

**Fig. 1:**
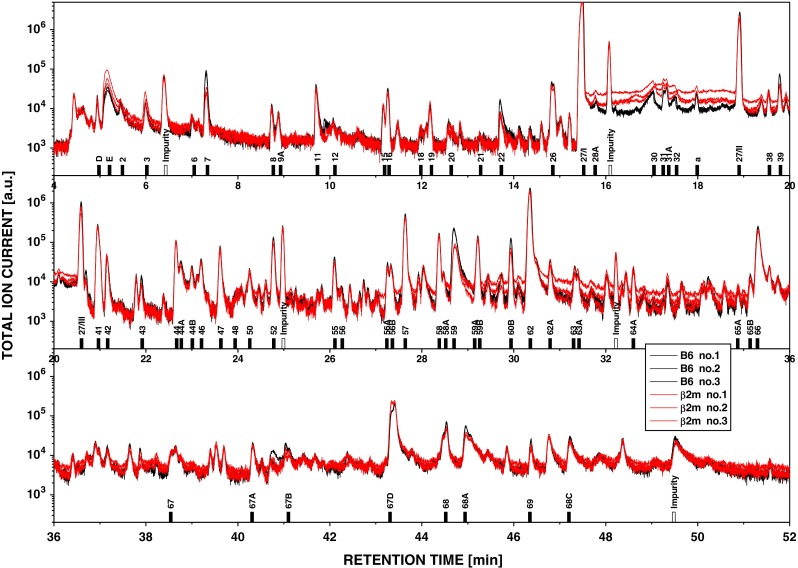
Gas chromatography/mass spectrometry of mouse urinary volatiles. Three chromatograms of aliquots of the same mixture of B6 (*black traces*) or B6/β_2_m^−/−^ mice (*red traces*) are shown. The total ion current is plotted on a logarithmic scale against the retention time. The figure gives an impression of the reproducibility of the tree measurements/sample. There is a nearly complete overlap between the chromatograms of the two samples indicating no qualitative differences. Some components (see for example peak for compound #7) show a small quantitative difference in abundance between the B6 and B6/β_2_m^−/−^ mice.* Numbers* below peaks refer to components, which can be looked up in the electronic supplement (Table S1) of Röck et al. [33] (unpublished data of Röck and Overath)

The problems encountered in the search for MHC-dependent volatiles can be exemplified by a study performed in our laboratory [33]. Urinary volatiles from groups of 15 individual male mice from a reference strain (B6J), two sub-strains of this strain (B6NCrl and B6F), a β_2_m-knockout strain congenic to B6J but lacking MHC class I molecules expressed on the cell surface (B6/β_2_m^−/−^J) and two unrelated inbred strains, BALB/cCrl and DBA/2Crl, were analyzed. The former four strains carry the H2^b^- the latter two strains share the H2^d^-haplotype. Enumerating 69 components quantitatively and up to 200 constituents qualitatively led to the following conclusions: Firstly, no qualitative differences were observed between the three B6 sub-strains or the B6/β_2_m^−/−^J mutant. However, five components present in B6 and BALB/c mice were absent in DBA/2 mice. Secondly, the individual variability in abundance within each group of mice was low for some components but high for others (standard deviation of the mean between 18 and 308 %). This variability implied that the odor bouquet varies between the individuals of the same strain. At least over several weeks, these individual odor profiles were relatively stable. Compared to strain B6J, the number as well as the magnitude of statistically significant quantitative differences increased in the order B6J ≈ B6/β_2_m^−/−^J ≪ B6F < B6NCrl < BALB/cCrl < DBA/2Crl. Most importantly, wild-type B6J and the B6/β_2_m^−/−^J mutant expressing no functional MHC class molecules differed less than strain B6J compared to its sub-strains. Figure 1 compares the volatile profiles obtained from mixtures of equal aliquots of urine sampled from 14 B6J mice (black) to that from 14 B6/β_2_m^−/−^J mice (red) by gas chromatography/mass spectrometry. In such mixtures, the individual variation was leveled out and no qualitative and only minimal quantitative differences are apparent.

In summary, urinary volatiles vary in abundance in individuals within a mouse inbred strain. This variability increases between sub-strains of the same strain and is expected to be substantial between congenic strains or wild-type versus mutant strains, particularly if they are used for scent analysis many years after their derivation. In contrast, the negligible variation between the B6J and B6/β_2_m^−/−^J mice, which reflects the rigorous breeding programs at the Jackson Laboratories, does not support an influence of MHC class I gene products on the composition of urine scent.

## The search for MHC-dependent peptides

The MHC is characterized by an extreme polymorphism, which is illustrated by transplant rejection between individuals of the same species. This polymorphism provided the initial stimulus for the proposal that the variability in this genomic region was somehow translated into an individual-specific olfactory signature, which allowed mice to identify their conspecifics. The highest polymorphism resides in the variable structure of the MHC class I and II glycoproteins. It is very difficult to envision a mechanism that explains how variable MHC protein structure itself has a direct effect on the composition of volatiles in body odor or urine, which comprise very diverse sets of secondary metabolites, yet indirect effects or influences of linked genes remain possible. In contrast, the idea that characteristic peptide ligands derived from the respective MHC class I molecules are released in urine and then function as signaling molecules would provide the required link between protein structure and behavior [4, 25]. Proof for this hypothesis entails three items of evidence: Firstly, MHC-derived peptide ligands must be present in mouse urine at functionally relevant concentrations. Secondly, because peptides are non-volatile, they must be taken up by sniffing into the nose of a mouse and then stimulate chemoreceptive sensory neurons. Thirdly, the MHC-derived peptides must elicit a specific behavioral response such as the pregnancy block described above at physiologically relevant concentrations.

Partial analysis of the urinary peptidome in several mouse strains by mass spectrometry has yielded no evidence for the occurrence of MHC class I-dependent peptides, the relevant criteria being peptide size, presence of the known anchor residues, and the exclusive existence in mice with the corresponding H2 haplotype [34]. In an alternative approach, we took advantage of a transgenic mouse strain that expresses chicken ovalbumin (OVA) under the control of the chicken β-actin promoter [35]. Despite the widespread occurrence of OVA in all organs and the good binding of the OVA-derived peptide SIINFEKL to H2-K^b^ molecules, SIINFEKL-H2-K^b^ complexes could only be detected in cells with dendritic morphology in an analysis of tail sections of B6/OVA^+^ mice [35]. These findings argue against the assumption that MHC-associated SIINFEKL is massively over-expressed throughout the body. Using a sensitive immunological assay, it was possible to detect SIIN**F**EK**L** (anchor residues in bold) at the extremely low concentration of 4 × 10^−12^ M in urine. This peptide was absent in urine of the wild-type B6 (β_2_m^+/+^) and ovalbumin-expressing, β_2_m-negative mice (B6/OVA^+^/β_2_m^−/−^) and therefore found to be dependent on the expression of both ovalbumin and MHC class I molecules.

**Fig. 2:**
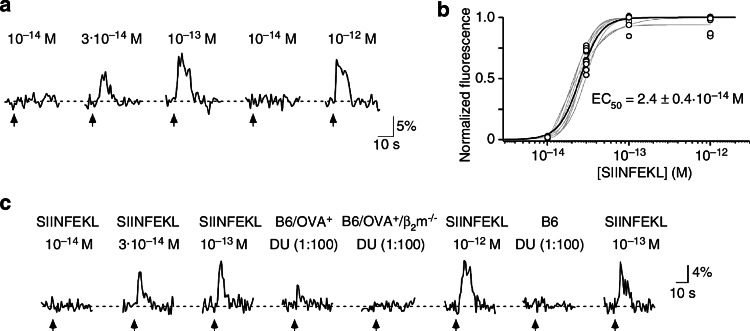
Analysis of peptide and urine-evoked Ca^2+^ responses in intact VSNs. **a** Example of somatic Ca^2+^ recordings from a single VSN to different concentrations of SIINFEKL. A response is defined as a stimulus-dependent deviation in Ca^2+^ fluorescence that exceeds twice the standard deviation of the mean of the baseline fluorescence noise. **b** SIINFEKL dose–response curves (*grey*) each recorded from a single VSN (*n* = 12 cells, eight slices). Relative changes in peak fluorescence (Δ*F*/*F*) induced by a given stimulus were normalized to the maximum peak response measured in a given VSN to construct a dose–response curve. The *black curve* represents the mean dose–response curve; EC_50_ value and SD are indicated. **c** Comparison of Ca^2+^ responses in a single VSN to various concentrations of SIINFEKL and urine (DU, diluted 100-fold to simulate the decrease in concentration in the VNO duct) obtained from B6/OVA^+^, B6/OVA^+^/β_2_m^−/−^, and B6 mice. This figure is taken from Sturm et al. [34], where further details can be found. Reprinted by kind permission of Trese Leinders-Zufall

If one assumes that SIINFEKL represents between 0.1 and 10 % of the H2-K^b^ ligands, the total urinary concentration of all MHC-dependent peptides may add up to 10^−10^–10^−8^ M. Because MHC class I molecules are considered to present thousands of different ligands, individual peptides are expected to be present at very low concentrations (≤10^−12^ M). Figure 2 shows that there are vomeronasal sensory neurons (VSNs) that can be specifically stimulated to saturation by synthetic SIINFEKL in the range of 10^−14^–10^−13^ M. Remarkably, such VSNs could detect the presence of SIINFEKL in 100-fold diluted urine of OVA^+^ mice, yielding an estimate for the peptide’s concentration close to that determined by the immunological assay. Taken together, the results with the model peptide fulfilled the first two criteria listed above [34]. However, the functional relevance of a urinary peptide concentration of 10^−12^ M has so far not been demonstrated in a behavioral context.

In addition to MHC ligands, urine could contain peptides derived from the MHC class I or II polypeptide chains themselves, which would reflect the polymorphic primary sequence of the respective haplotype. In fact, rat urine contains fragments of MHC class I molecules at a concentration of about 1 nM [36, 37] (confirmed by us for mice). However, such peptides were not detected in our partial analysis of the urinary peptidome.

**Fig. 3:**
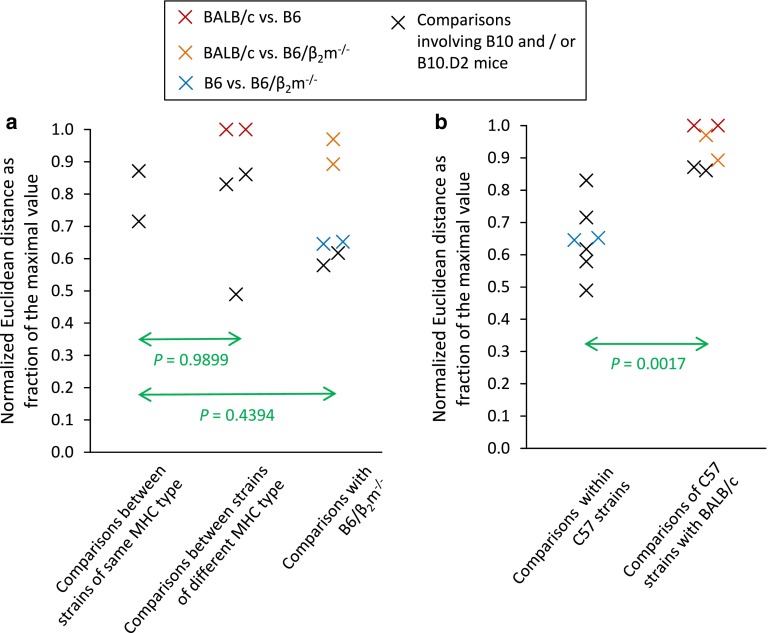
Evolutionary relatedness has a much stronger impact on the urinary peptidome than the MHC. Urinary peptides of five inbred mouse strains were analyzed by mass spectrometry (data from reference [34]), and the normalized Euclidean distances of hundreds of peptide intensities were calculated according to Natsch et al. [62]. The results are depicted as a fraction of the maximal Euclidean distance obtained in the respective experiment. The experiments included BALB/c mice (*H2* ^*d*^) and four strains of the C57 lineage: B10 (*H2* ^*b*^), B10.D2 (*H2* ^*d*^), B6 (*H2* ^*b*^) and B6/β_2_m^−/−^ (*H2* ^*b*^, but lacking functional MHC class I molecules) yielding ten pair-wise comparisons, which are all depicted in both panels (**a**, **b**). BALB/c, B6 and B6/β_2_m^−/−^ mice were each analyzed in two biological replicates yielding two data points for the respective comparisons, which are indicated with* colored crosses*. These duplicate Euclidean distances were joined to their respective mean value before performing the two-tailed, heteroskedastic Student’s *t* tests. **a** The differences in the urinary peptide pattern are independent of the MHC. **b** Ordering the data depicted in **a** according to the evolutionary relatedness readily reveals a highly significant difference in the urinary peptidome of BALB/c mice versus the strains of the C57 lineage. A small proportion of this difference is due to SAV peptides and MUP20-specific peptides (see reference [34] for details). However, even when removing the latter two peptide groups from the data set, BALB/c urine clearly separates from C57 urine increasing the *p* value merely from 0.0017 to 0.0020. Consequently, most of the differences in the urinary peptidome remain unexplained [48]

In theory, urinary peptides occurring in association with a certain MHC type might also result from indirect effects of the MHC or linked genes apart from MHC peptide ligand processing. Pairwise comparisons of the urinary peptide pattern of five mouse strains (H2^b^ and H2^d^ haplotypes) by calculation of the Euclidean distances for several hundred peptides show no differences that correlate with the MHC (Fig. 3a). Hence, there appears to be no global influence of the MHC on the urinary peptidome.

While MHC-dependent peptide ligands turned out to be below the detection limit in mass-spectrometric analysis, this technique revealed the presence of a class of urinary peptides which conformed in size and anchor residues to ligands of several H2 class I alleles (K^b^, D^b^, K^d^, or L^d^) of the mouse strains analyzed. However, the occurrence of these MHC motif peptides in urine was *MHC*-*independent*, that is, they were present in urine of both H2^d^ and H2^b^ mice including β_2_m^−/−^ mice. One of these peptides, TRVL**N**LGP**I**, and two of its extended forms (12 or 18 residues long) were shown to selectively stimulate a subset of VSNs at very low concentrations (10^−11^ M). Importantly, these MHC-independent peptides are present in urine in concentrations up to 10^−6^ M, showing that they are up to six orders of magnitude more abundant than the prototypic MHC-dependent peptide SIINFEKL [34].

In view of these observations, designing suitable assays for proving or disproving a role of the MHC-dependent peptides in stimulating behavioral responses is a real challenge. For example, a comparison of wild-type urine and urine from β_2_m mutant mice in the pregnancy block test may appear informative. However, rather than showing a differential response to the absence or presence of MHC-dependent peptides of very low abundance, the mice may recognize the difference in much more abundant peptides derived from the β_2_m protein, itself an established protein component of urine. A more valid approach could be to compare the extent of pregnancy failure between different inbred strains H2^x^/β_2_m^+/+^ and H2^y^/β_2_m^+/+^ versus H2^x^/β_2_m^−/−^ and H2^y^/β_2_m^−/−^. If MHC-derived peptides play a role, the pregnancy block should at least be significantly reduced in the mutant situation because these peptides will neither contribute to memory formation in the initial mating nor to the subsequent recognition of the strange male or its urine.

As mentioned above, the pregnancy block could be elicited by synthetic class I peptides harboring non-mating male MHC motifs but not with mating male motifs [25]. In the case of H2-K^d^-specific peptides used in this study only two (S**Y**FPEITH**I** and S**F**VDTRTL**L**) were derived from mouse proteins while the third (S**Y**IPSAEK**I**) corresponded to a sequence in the *P. berghei* circumsporozoite protein. In the case of H2-D^b^-specific peptides (AAPD**N**RET**F** originating from erroneous peptide sequencing, ASNE**N**MET**M** from influenza A/PR8/34 nucleoprotein, FAPG**N**YPA**L** from the SV 40 nucleoprotein), all three were not encoded in the mouse genome. Surprisingly, and in spite of the fact that the urine of both H2^b^ and H2^d^ mouse strains contains ample MHC-independent peptides with either MHC motif, peptides were judged as familiar (mating male) or strange (non-mating male) not according to the peptides’ (mostly non-mouse) protein origin but according to their MHC-motif. The H2-K^d^ peptides supplied to male BALB/c urine behaved as familiar in BALB/c (H2^d^)-mated BALB/c females but as strange in B6-urine of B6 (H2^b^)-mated BALB/c females. Accordingly, the H2-D^b^ ligands acted as familiar in B6-urine of B6-mated but as strange in BALBc-urine of BALB/c-mated BALB/c females. However, the relevance of these experiments for individual recognition under natural conditions is problematic considering the non-physiologically high concentration of 10^−4^ M for each peptide used in these studies. Finally, the requirement of urine as an additional stimulus in these experiments requires clarification, because in the first study [25], three peptides were applied within urine while a more recent paper described an effective pregnancy failure using only one of these peptides in the absence of urine [38]. From a theoretical point of view, it appears very puzzling that an MHC peptide ligand should exert a biological effect independent of its biochemical context. In practice, this would imply for example that the pregnancy block effect is triggered when the female mouse encounters an unfamiliar MHC motif peptide, for example in strange female urine or food (note that such peptides will occur just by chance in any biological sample containing proteins [34]). In fact, it is known that strange female urine does not elicit the pregnancy block effect, whereas urine from androgenized females is effective [39].

## Genomic variability of the urinary peptidome independent of the MHC

Apart from differences in MHC-dependent peptides, the urinary peptidome can be expected to be modulated by genomic variability, which may manifest itself in at least three ways. Firstly, single-nucleotide variations (SNVs) in structural genes can lead to single amino acid variations (SAVs) in peptides derived from proteolysis of the respective polypeptides. Secondly, nucleotide insertions or deletions in protein-coding exons are anticipated to alter the sequence or presence of urinary peptides. Thirdly, mouse strains differ in the expression of certain genes, which directly and possibly also indirectly affect the composition of the urinary peptides.

Out of 639 peptides identified in a partial peptidomic analysis of mouse urine, at least 47 turned out to be affected by SAVs and are therefore encoded only in some *Mus musculus* subspecies or laboratory strains (see Supplementary Table S8 in reference [34]). Such SAVs occur even within related inbred strains, for example in the C57BL/6 lineage, but are particularly frequent between different *Mus musculus* subspecies. Specifically, mass spectrometric analysis showed that one peptide encoded by the *Ces1*c gene and 24 peptides encoded by the *Serpina3k* gene differed between BALB/c and C57BL/6 mice in one or sometimes two amino acid residues. One such SAV peptide pair lacking an MHC motif was tested at a concentration of 10^−11 ^M for recognition by VSNs. Some VSNs responded only to the B6-form of the peptide (IVIYHTSAQSIL) but not to the BALB/c-form (FVIYHTSAQSIL) whereas others showed the reverse activation pattern or recognized both peptides. Importantly, the concentration of SAV peptides in urine is similar to peptides with MHC motifs (≤10^−6^ M), suggesting that they may be of functional significance for mice to identify conspecifics [34].

Major urinary proteins (MUPs) are considered to be important signaling molecules in the communication of mice [40, 41]. Because MUPs are highly polymorphic proteins encoded by several paralogous genes in *M. musculus*, one can expect strain specific variability in the derived urinary peptide spectrum [42]. In fact, 115 peptides (18 %) in our analysis of one HPLC fraction of mouse urine corresponded to MUP sequences establishing MUP-derived peptides as major peptides in this fluid although the percentage of MUP-derived peptides might be different in other HPLC fractions. Strain-specific qualitative differences in urinary MUP peptides are likely to exist within *M. musculus* but could not be specified so far. A major difficulty for the experimental demonstration of such peptides is the incomplete strain-specific MUP protein sequence information. However, qualitative differences clearly occur between *M. musculus* and the closely related species *M. macedonicus*. Out of the 115 *M. musculus* MUP peptides, 58 are not encoded in the single MUP gene of *M. macedonicus*. Between these two sympatric species, the differences can be as high as six amino acid residues per urinary peptide [34, 43]. Within *M. musculus*, the expression of MUPs differs at least in quantitative terms. For example, the proportion of MUP20 (also known as darcin) among all MUPs is only <0.5 % in male BALB/c but about 15 % in male B6 mice [41]. Consequently, unique peptides from MUP20 were found to be more frequent and more abundant in male urine of B6 than BALB/c origin. Finally, because females do not express MUP20, derived peptides are predicted to be sex-specific [34].

Apart from MUPs, there are also other proteins affected by genes with allele-specific occurrence or function. Correspondingly, 21 peptides derived from meprin A α, a urinary metalloendopeptidase, could be identified in urine of C57 and BALB/c mice [34]. The urine of strains with the H2^k^ haplotype, e.g., C3H or AKR, lacks meprin A α, and the nonfunctional *mep1a* allele is genetically linked to the H2^k^ haplotype [44–46]. Mice deficient in this protein will not only lack the peptides encoded by *mep1a* but could also have a changed proteolytic cleavage pattern of urinary peptides. The effect of meprin A α deficiency should be considered when comparing mice of the H2^k^ haplotype with MHC congenic individuals in potentially MHC-related behaviors such as mating. Specifically, mating between B6-H2^b^ and B6-H2^k^ strains (see above) could be influenced by the difference in the *meprin A α*-gene expression as a consequence of the fact that congenic strains can differ over large parts of chromosome 17 harboring many genes in addition to MHC class I and II genes [47] (see Fig. 4–1 in reference [48]).

In summary, the urinary peptide composition reflects a multitude of diverse genetic variations, which are manifest in differences in both peptide sequence and abundance (see Table 1 for examples of direct and indirect genomic effects). Qualitative and quantitative differences in the peptide pattern will increase with decreasing evolutionary relatedness (Fig. 3b) in agreement with observations made for the composition of urinary volatiles. Given the fact that nasal neurons recognize and differentiate numerous peptides with exquisite sensitivity, the urinary peptide pattern represents a real-time sampling of the genome for chemosensory assessment by other individuals [34, 49].

**Table 1:** Genomic variations known or expected to influence the mouse urinary peptidome

Type of variation (direct effects)	Effect of variation on peptides in urine	Affected proteins	Example for peptide in urine [34]		Evidence for peptide in urine [34]	
			Standard form (C57BL/6 mice)	Varying form	Standard form	Varying form
SAV	Exchange of single amino acid at single place	Most, e.g., serine protease inhibitor A3K, further examples in Supplementary Table S8 of reference [34]	**I**VIYHTSAQSIL	**F**VIYHTSAQSIL (BALB/c mice)	Yes	Yes
SAV changing posttranslational modification (PTM)	New PTM or loss of PTM at site of SAV	Some, e.g., serine protease inhibitor A3K	PAV**C**FNRPFL | cysteinyl	PAV**H**FNRPFL (BALB/c mice)	Yes	Yes
Multiple amino acid variations (highly polymorphic genes)	Exchange of single amino acids at multiple places close to each other	Few, e.g., MUPs	**T**I**P**KTDYDN**FL**M and several variants thereof, e.g. **S**I**L**KTDYDN**YI**M	Only **T**I**P**KTDYDN**FL**M (*Mus macedonicus*)	Yes	Not yet
Insertions and deletions	Peptide containing at least one additional amino acid or lacking at least one amino acid	Few	n.a.	n.a.	Not yet	Not yet
New stop codon	C-terminally shortened peptide; absence of certain peptides	Few	n.a.	n.a.	Not yet	Not yet
Inactive allele	Absence of certain peptides	Few, e.g., meprin A α (Fig. 5 of reference [44])	FQGDILLPR	Peptide missing (C3H/He mice)	Yes	No demonstration of deficiency until now
Quantitative differences in expression	Changed concentration	Some, e.g., MUP20 (reference [41])	VEYIHVLENSL at high concentration	VEYIHVLENSL at low concentration (BALB/c mice)	Yes	Yes
Peptide sequence variation restricted to protease cleavage site	Splitting of peptide into two parts (gain of cleavage site); fusion of two peptides (loss of cleavage site); altered sequence at cleavage site	Some?	n.a.	n.a.	Not yet	Not yet

Type of variation (indirect effects)	Effect of variation on peptides in urine	Affected proteins	Example for peptide in urine [34]		Evidence for peptide in urine [34]	
			Standard form (C57BL/6 mice)	Varying form	Standard form	Varying form
Sex-specific expression	Several peptides occurring only in urine of one but not the other sex; sex-specific quantitative differences	Few, e.g., MUP20 (qualitative difference [41]); other MUPs (quantitative difference [67])	VEYIHVLENSL (males)	Peptide missing (females)	Yes	No demonstration of deficiency until now
MHC-dependent effect	Low abundant urinary peptides occurring in mice of one MHC type but not in mice of other MHC types	Most?, caused by differential binding of peptides to MHC molecules	SIINFEKL (B6/OVA^+^ mice)	Peptide missing (B6/OVA^+^/β_2_m^−/−^ mice)	Yes	Yes (demonstration of deficiency)
Variation in activity or specificity of protease	N- and/or C-terminally extended or shortened peptides; new peptides in urine; absence of certain peptides	Many, e.g., caused by differential activity of meprin A α (Fig. 5 of reference [44])	n.a.	n.a.	Not yet	Not yet
Variation of gene “A” influences expression of conserved gene “B”	Changed concentration	Some?	n.a.	n.a.	Not yet	Not yet
Variation of gene influencing PTMs in products of other genes	Altered PTMs could occur in many peptides at the same time	Some?, e.g., caused by differential activity of kinase or lysine acetyltransferase?	n.a.	n.a.	Not yet	Not yet

This table contains only selected genomic variations known or expected to influence the urinary peptidome without claiming that the list is comprehensive. The capability of VSNs to discriminate single amino acids in peptides is well documented, and length variations of the same core peptide also seem to be recognized by some of these neurons [34, 52]. In contrast, it remains to be tested whether VSNs distinguish between peptides with and without posttranslational modifications (PTMs).* n.a*. (not available) indicates that an example has not yet been demonstrated experimentally

## The olfactory dilemma of peptides with MHC motif

Although in the case of mice, MHC class I-derived peptides are present in urine and can be differentiated by some vomeronasal sensory neurons, their concentration is extremely low. Implicit in the original “MHC peptide ligand hypothesis” as proposed by Boehm [4] is that these low-abundant peptides qualify as stimulants of a separate and specialized sensory pathway that connects the activation of specific receptors or cells in the periphery with a distinct sensory perception or behavior [50, 51]. It was assumed that only the MHC motif is important for MHC peptide ligand recognition by VSNs [4, 25, 38]. If this hypothesis were true, one could now rule out an influence of MHC-dependent peptides on olfaction because the sum of the urinary concentrations of all peptides with a specific MHC motif is clearly dominated by the MHC-independent MHC motif peptides.

However, VSNs can discriminate MHC-dependent peptides from MHC-independent MHC motif peptides on an individual basis, i.e., by the additional recognition of amino acid residues apart from the MHC anchors (Fig. 2; references [34, 52]). The VSNs detecting MHC-dependent peptides seem to overlap with those detecting MHC-independent ones. Single MHC-dependent peptides can be discriminated from other, frequently much more abundant peptides by the combinatorial activation pattern of VSNs, but VSNs exclusively designed to respond to MHC-dependent peptides are unlikely to exist because the MHC-dependent peptides lack specific structural features not occurring in MHC-independent urinary peptides.

Hence, although MHC-dependent peptides might marginally contribute to the general peptide pattern sensed by the olfactory systems, the crucial question remains: how should the mouse brain interpret the MHC-dependent information as an expression of the immune system and not just as one out of many other types of genomic variation (for a more detailed discussion refer to reference [48])? Therefore, we consider it highly unlikely that animals alter their behavior specifically according to the MHC-type of other individuals. Instead, behaviors might be adapted with respect to overall genomic relatedness. Avoidance of mating with closely related individuals will inherently reduce the number of MHC homozygous offspring because unrelated mating partners are unlikely to share the same MHC alleles due to the extreme MHC polymorphism in most natural populations.

## MHC and behavior in fish and man

Individual male sticklebacks (*Gasterosteus aculeatus*) are considered to release MHC-dependent odors that provide information about the degree of diversity of their MHC genes. These olfactory cues are postulated to be used by females during mating in order to achieve an optimal number of MHC alleles in their offspring. Gravid female sticklebacks were exposed in a flow channel to two different water sources, each originating from a tank harboring a single male. Under these conditions, females appear to compare their own set of MHC class IIB alleles with those of potential mating partners and show preference for the scent of the male with the optimal complement of alleles. Using in silico predictions, 9-mer peptides with mouse or human MHC class I motifs (here considered to represent both MHC class I ligands and trimmed MHC class II ligands) were selected from stickleback protein sequences. Such peptides were then synthesized. The potential mate choice of females could be manipulated by either mixtures of the stickleback peptides or the synthetic mouse class I peptides previously used in the pregnancy block experiments (see above). The study suggests that peptides with MHC class I motifs are recognized by the fish olfactory system [53] and considered to modify mate choice. Although fish MHC proteins might have specificities for peptide ligands similar to that of mouse homologues [54], it should be pointed out that MHC binding motifs usually differ strongly even for different alleles of the same species. It is this MHC allele-specific motif that contains information on the MHC type of an individual and not the presence of MHC peptide ligands per se. Therefore, the experiments in sticklebacks cannot resolve whether the applied peptides are behaviorally interpreted with respect to MHC type or whether the peptides merely act as signals of foreignness, which could be simulated by the addition of any peptide detectable by the olfactory system. Furthermore, it remains to be demonstrated whether the fish release MHC-dependent peptides in relevant concentrations into the water (with urine?) and thereby elicit the expected behavioral reaction.

The proposition that mate selection in humans is influenced by the HLA region by way of odorants dates back to studies in which females had to judge the pleasantness of male body odors on worn T-shirts, demonstrating that there was a preference for the scent of HLA-dissimilar persons [55]. Ensuing studies on odor preference and marital choice gave conflicting results [56]. Using a large number of genome-wide SNP markers in the HapMap II data set, a recent study suggested that there is a tendency for MHC-disassortative mating in a sample of European-American couples from the Mormon community [57, 58]. The authors consider two explanations for this effect: discrimination by MHC-mediated scent or selection of the spermatozoa by the female oocyte (post copulatory sexual selection, see reference [59] for review). In contrast, Yoruba African spouses showed no significant pattern of similarity/dissimilarity across the MHC region compared to the entire genome [57]. Moreover, analyzing the same HapMap II data set and an independent set of European-American mates, Derti et al. [60, 61] came to the conclusion that HapMap genotypes do not confidently support a role of the MHC locus in human mate selection. Therefore, evidence for an influence of the MHC on mate choice in humans remains controversial.

In humans, secretions by the axillary glands are considered to be of decisive importance for providing behaviorally relevant odors. The initial sweat is without scent but highly odorous components are produced by skin bacteria. Using sweat collected from 12 HLA-typed families, a diverse class of volatile, highly odorous carboxylic acids was released from their glutamine conjugates by *N*_α_-acyl-glutamine-aminoacylase from *Corynebacterium striatum* Ax20. Quantification of the methyl esters of the panel of acids by high-resolution gas chromatography/mass spectrometry allowed the comparison of the pattern of the analytes by calculating their normalized Euclidean distance. The distance was lowest between samples taken from the same individual. A much higher distance was observed between subjects of the same family, but there were no differences between siblings sharing none, one, or both HLA-alleles (Fig. 1 in reference [62]). Therefore, the pattern of glutamine conjugates of volatile carboxylic acids in the human axillae appear not to be influenced by genes in the HLA complex [62].

Importantly, a study in humans [63] leads to exactly the same conclusion as the analysis of urinary mouse volatiles: There is individual variation in homozygotic twins/inbred mice yet variation is much larger between unrelated humans/different inbred strains. The distance of samples increases in the order (same individual, same day) ≤ (same individual, different day) < (homozygotic twins) ≪ (unrelated individuals) [63]. The mouse studies [32, 33] clearly indicated a strong genetic influence, which is in agreement with the observations in humans, although the human study [63] did not exclude maternal and environmental effects on body odor.

Humans discriminate individuals by vision, they lack a vomeronasal organ, and close sniffing is generally restricted to sexual interaction. Nevertheless, a recent study claims that females can smell the difference in peptides with self versus non-self HLA class I motifs (SLLPAIVEL for HLA-A2 and KYPENFFLL for HLA-A24) applied to their arm pits. Moreover, certain brain regions are specifically activated when these peptides (at 25 μM in buffer) are directly applied to the nose in an air stream generated by an olfactometer, demonstrating that peptides can activate sensory neurons in the human olfactory epithelium [64]. However, the authors only applied spectrophotometry to demonstrate the aerosolization of a synthetic MHC peptide ligand in the olfactometer [65]. They report neither a peptide sequence-specific assay nor a negative control for the potential contamination of the analytical devices with proteins and peptides derived e.g., from shed dead skin cells (a common problem in proteomic laboratories). Whether sweat secreted into the armpits contains HLA-derived peptides at relevant concentrations and whether non-volatile olfactory cues can reach the human olfactory epithelium by sniffing remains to be shown [66].

## The scent of the immune system: an illusion?

The main conclusion from this article is that individual variation in volatiles and peptides, which becomes larger with a decrease in the degree of relatedness, can explain attraction or aversion in behavioral contexts such as mating, kin and parent-progeny recognition, as well as inbreeding avoidance without invoking a specific influence of the MHC class I and II genes. As pointed out in a recent editorial [49], this variability may provide “a universal mechanism of olfactory assessment of genetic individuality even for animals that do not possess an MHC”. We realize that this reinterpretation of studies by many laboratories over many years might raise opposition. For example, in a review by Kwak et al. [32], the evidence that MHC variation influences volatiles of mouse urine and body odors obtained by behavioral and Y-maze experiments with F_2_-segregants of congenic strains is considered to be incontrovertible and, therefore, chemical studies are proposed to have so far missed important information. The proverbial needle in the haystack might indeed have been missed for the volatiles, but we think that the haystack—MHC-independent variation of olfactory cues—is sufficient to signal genomic identity. For reasons detailed above, we find the results of pregnancy-block studies using peptides with MHC motifs puzzling and in need of further experimentation taking into consideration the very low concentrations of MHC-dependent peptide ligands now demonstrated in urine.

We favor the view that sensing the gestalt of the urinary peptidome as well as variability in MUPs and volatiles provides enough critical information for individual recognition without the requirement for the apparently minute MHC-dependent differences. Thus, the reason for the evolution of the extensive polymorphism of the MHC class I and II proteins remains with their immunological function: combating the evasive strategies of pathogens.
